# Comparing compliance with the WHO surgical safety checklist and complication rates in gynecologic surgery between day and night shifts

**DOI:** 10.1007/s00404-022-06599-w

**Published:** 2022-05-27

**Authors:** Bekos Christine, Bodner-Adler Barbara, Sonja Zehetmayer, Umek Wolfgang

**Affiliations:** 1 Division of General Gynecology and Gynecologic Oncology, Department of Obstetrics and Gynecology, Medical University of Vienna, Vienna, Austria; 2 Center for Medical Statistics, Informatics and Intelligent Systems, Vienna, Austria

**Keywords:** Perioperative management, Postoperative complications, Checklists, Gynecologic operations, Shifts, Working time

## Abstract

**Purpose:**

At least half of surgical complications can be avoided by using surgical checklists. However, universal implementation and compliance have been reported as being variable. Patients undergoing urgent surgical intervention are at increased risk for complications. The aim of this study was to evaluate the checklist compliance together with the complication rate during day and night shifts in a European University hospital.

**Methods:**

51 and 52 consecutive patients who had surgery during day and night shifts were included. The primary outcome measures were compliance and completeness of the WHO safety checklist. The occurrence of postoperative complications was investigated.

**Results:**

The analysis included 103 surgical procedures. The mean compliance rate of use was 93% and the mean completeness rate was 22%. After operations were broken down by day or night shift, we found that checklists were less often available in night shifts compared to day shifts. The completeness of the checklist and the occurrence of postoperative complications did not differ between day and night shifts.

**Conclusion:**

This study reports worse checklists availability in night shifts when compared to day shifts, but complication rates did not increase. Further studies are warranted to investigate postoperative complication rates together with checklist compliance in day versus night shifts.

**Supplementary Information:**

The online version contains supplementary material available at 10.1007/s00404-022-06599-w.

## Introduction

Surgery is performed in great numbers each year to save lives and improve quality of life. In Austria, 1.1 million surgeries were performed in 2020 [1]. Surgery implies that the risk of complications and rates of complications related to the surgical procedure vary, occurring in 4.3% [2] to 13% [3] of non-cardiac surgical procedures. Surgical complications are a major cause of morbidity and mortality and also pose a major financial burden to patients and providers [4, 5].

Wrong-side surgery, omission of antithrombotic measures, omission of prophylactic antibiotics, and mix-up of blood groups are among the most consequential preventable complications [4, 5].

Patients undergoing urgent surgical intervention are at even higher risk ofr complications and death [6]. Even routine surgery requires the complex coordination of surgeons, anesthesiologists, nurses, and support staff to provide timely and effective care, but heightened patient acuity and time pressure increase the potential for critical errors and omissions in established standards of care.

Scientific studies have shown that the use of a checklist can save lives and reduce the morbidity as a result of improved patient outcomes including reduced infections, wound rupture, respiratory complications, bleeding, and blood transfusions [7].

Surgical checklists have been developed to decrease perioperative complication rates and to increase the safety of surgical care. A number of checklists have been proposed, but the most widely known and implemented is the WHO surgical safety checklist (SSC) [8]. This intervention was part of the safe-surgery-saves-lives-challenge, aiming to improve surgical care safety around the world by ensuring adherence to proven standards of care.

However, the WHO surgical safety checklist advances standard perioperative checklist practices in several key ways. First, it is administered in the operation room (OR), not in the preoperative area as has often been the case. Second, it is administered at three strategic points: on patient arrival but before any intervention (“sign in”); after induction of anesthesia, immediately before surgical incision (“time out”); and before team members or the patient leaves the OR (“sign out”). Finally, it is specifically designed to promote communication and teamwork in the OR.

Despite the benefits associated with the use of checklists in surgery, universal implementation and compliance have been reported as being variable and inconsistent [9].

Cesarean sections performed during night shift are associated with longer duration of surgery and an increased risk of maternal morbidity [6]. Data regarding compliance with surgical checklists comparing day shifts and night shifts and the related complication rate are missing.

Our hospital has adopted the WHO surgical safety checklist with minimal adaptions for local practicability in 2011 and made its use mandatory in all operating rooms. This study set out to compare the compliance with the WHO surgical safety checklist and complication rates in surgical cases performed during day shifts versus those performed during night shift.

## Methods

We conducted a cross-sectional study at the Department of Obstetrics and Gynecology, Medical University of Vienna. We retrospectively assessed the compliance with the WHO surgical safety checklist, its completeness, and the complication rates in gynecologic surgical cases, comparing day shifts with night shift.

The study was approved by the Ethics Committee of the Medical University of Vienna (IRB approval number: 1696/2019). The ethics committee waived the requirement to obtain informed consent from patients.

We included a random sample of 103 patients who underwent gynecologic surgery between 04/2016 and 10/2018, using a computer-generated randomization list.

We compared the charts of 51 patients who had had surgery during day shifts with 52 patients who had had surgery during night shifts at the Department of Obstetrics and Gynecology. Patients operated during day and night shifts included both elective and acute surgeries.

For physicians in our hospital, the day shift is the time between 8am and 4pm and the night shift is the time between 4pm and 8am of the following day.

In our hospital, the WHO surgical safety checklist is a paper form which is added to the patient’s chart on entry of the patient into the OR area. It is read out loud at sign in, time out, and sign out in the presence of the entire surgical team, including surgeons, anesthesiologists, nursing and auxiliary staff and is ticked off by a scrub nurse. It then becomes part of the patient’s paper chart.

The primary outcome measure in our study was the compliance rate with the WHO surgical safety checklist. We defined compliance based on whether the checklist was part of the patient´s chart. We defined completeness as “complete”, if all items of the checklist were checked; “incomplete”, if one of more items were left unchecked.

The secondary outcome measure was the occurrence of any postoperative complication according to the Clavien–Dindo classification (CDC) [10]. Type of surgery (classified as small, medium, or major), duration of surgery in minutes, day versus night shift, and type of anesthesia (general versus regional versus local anesthesia) were recorded for all patients.

Minor surgeries were classified to include hysteroscopies, dilation and curettages, conizations, marsupializations, and abscess drainages. Medium surgeries included laparoscopies, surgery of the breast, lymph node excisions and laparotomies of less than 300 min duration. Major surgeries included all surgeries lasting longer than 300 min, typically debulking surgeries for ovarian cancer or extensive endometriosis.

We reviewed all patients’ charts until discharge and beyond to include complications which occurred after discharge from the hospital. In case of a postoperative complication, patients usually return or are referred to the hospital where surgery was performed, which guarantees that little to no complications are lost to follow-up.

We used descriptive statistics to describe availability and completeness of the WHO surgical safety checklist as part of the patient´s chart. We present categorical variables as absolute and relative frequencies; We present metric variables as median and range (minimum, maximum) because none of these variables was normally distributed. Metric variables were analyzed using Wilcoxon signed-rank test, and categorical variables were analyzed using Chi-squared test or Fisher´s exact test if sample size was small.

A *p* value of < 0.05 was considered statistically significant. Statistical analyses were calculated using SPSS 27.0 for MAC (SPSS 27.0, IBM Inc., Armonk, NY). The database with patients’ records was anonymized and de-identified prior to analysis.

## Results

The analysis for this studywa based on 103 surgical procedures at the Department of Obstetrics and Gynecology, Medical University of Vienna. 51 procedures were performed during day shifts and 52 procedures during night shifts. Patients’ median age was 42 years, with a range from 12 to 89 years. 23% of all surgeries were classified as minor, 68% as medium, and 9% as major surgery, which is representative for all surgeries in our department. The median duration of surgery was 110 min (range 20–520 min).

98% of surgeries were performed under general anesthesia, 2% of surgeries were performed using spinal anesthesia and none under local anesthesia.

In 26% of cases one or more participants of the surgical team were exchanged, meaning that they left or joined at a later stage. During the day, surgical teams changed in 31% versus 21% during the night, a non-significant difference. Postoperative complications were observed in 17 patients (17%), and of these only 2 (1.9%) had severe complications (CDC≥3). There was no difference regarding the rate of complications between day and night shifts. For classification and frequency of postoperative complications, see Table 1.

**Table 1:** Classification and frequency of postoperative complications according to Clavien–Dindo

	*n*
CDC severity 1–2	
Sensory disturbance in one leg	1
Postoperative bleeding, treated with tranexamic acid	1
Re-admission for intravenous analgesics	1
Anemia requiring blood transfusion	7
Postoperative fever, treated with antibiotics	5
CDC severity ≥ 3	
Surgical evacuation of a hematoma under general anesthesia	1
Bowel resection with stoma creation	1

*CDC* Clavien–Dindo classification, *n* numbers

The overall compliance rate for the use of the WHO surgical safety checklist was 93%, meaning that a checklist as part of the patient chart was missing in 7% of all cases. Compliance was better during the day with 100% of checklists being part of the patients’ chart compared to 87% during night shifts. This difference was statistically significant (*p* = 0.013), but it did not result in a difference in complication rate.

However, only 22% of checklists were complete. In 78%, one or more items on the checklist were not ticked off. Completeness of the checklist did not differ between day and night shifts and was low in general.

In night shifts, major surgeries were less often performed, and revision operations were more frequent. Age was significantly lower in patients who had surgery during night shifts and duration of surgery was also significantly shorter. The comparison of day versus night shift is shown in Table 2.

**Table 2:** Patient characteristics broken down by day versus night shift

	Day shift (*n* = 51)	Night shift (*n *= 52)	*p* value
Checklist available [*n* (%)]	51 (100%)	45 (87%)	0.013^c^
Checklist completed [*n* (%)]	9 (18%)	14 (27%)	0.345^a^
Type of anesthesia [*n* (%)]			1.000^c^
General anesthesia	50 (98%)	51 (98%)	
Spinal anesthesia	1 (2%)	1 (2%)	
Team changes [*n* (%)]			0.269^a^
Yes	16 (31%)	11 (21%)	
No	35 (69%)	41 (79%)	
Type of surgery [*n* (%)]			0.041^c^
Minor	10 (20%)	14 (27%)	
Medium	33 (65%)	37 (72%)	
Major	8 (15%)	1 (1%)	
Revision surgery			< 0.001^c^
Yes	0	13 (25%)	
No	51 (100%)	39 (75%)	
Indication for surgery			< 0.001^c^
Sterility	5 (10%)	3 (6%)	
Suspicion of malignancy	28 (55%)	2 (4%)	
Bleeding	9 (18%)	19 (37%)	
Acute inflammatory disease^d^	1 (2%)	5 (10%)	
Acute abdomen	4 (8%)	7 (14%)	
Extrauterine pregnancy	0	8 (15%)	
Missed abortion	1 (2%)	8 (15%)	
Pelvic floor dysfunction	3 (5.9%)	0	
Admission [*n* (%)]			< 0.001^c^
Planned	49 (96%)	0	
Unplanned	2 (4%)	52 (100%)	
Age, years [median (min–max)]	48 (19–89)	36 (12–67)	< 0.001^b^
Duration of surgery, min [mean (SD]	166 (115)	93 (37)	0.002^b^
Postoperative complications [*n* (%)]			0.597^a^
Yes	7 (14%)	10 (19%)	
No	44 (86%)	42 (81%)	

*SD* standard deviation, *n* numbers

^a^Chi-squared test

^b^Wilcoxon signed-rank test

^c^Fisher’s exact test

^d^This category includes abscesses of the adnexa, Bartholin gland and the breast

## Discussion

In our study, we found a mean compliance rate with a mandatory checklist of 93%, albeit with a very low rate of completeness. We had defined compliance based on whether the checklist form became part of the patients´ chart and completeness as whether one or more items on the WHO surgical safety checklist were left unchecked. Completeness was only 22%, but did not differ between day and night shifts and was low in general. Compliance was significantly lower during night shifts than during day shifts.

An Australian study evaluating the compliance with the WHO surgical safety checklists in acute and elective procedure found similarly low rates of correct checklist administration. In that study, the checklist was used at the beginning of surgery in 99% compared to only 2% at the end of surgery. Especially, the completeness of the “sign out” part was low [11]. These findings are consistent with a recent study in British hospitals, which found that sign out was completed less commonly than the other domains [12]. A possible reason for the poor compliance with this domain is confusion about its proper timing which is defined as ‘before the surgeons leave the OR’. Unlike other domains, “sign out” it is not linked to a specific event in patient management.

Van Klei et al. reported a significant reduction in perioperative mortality when the WHO SSC was completed, OR: 0.44 (95% CI 0.28–0.70), but not when the SSC was incomplete [OR 1.09 (95% CI 0.78–1.52)], or noncompleted [OR 1.16 (95% CI, 0.86–1.56)] [13]. In our study, we did not evaluate the effect of compliance with the WHO SSC on patient outcome. However, we believe these previous studies provide data which support the contention that compliance impacts on safety.

We observed postoperative complications in 17 patients; two of these were severe complications equaling grade 3 or higher according to Clavien–Dindo. There was no difference in frequency of complications between day and night shifts. In an Iranian hospital, the implementation of surgical safety checklists reduced the incidence of any complication from 23 to 10% [14]. This study collected data on severe postoperative complications, but missed detection of mild complications such as urinary tract infections, necessity of blood transfusions and postoperative application of antibiotics, which are all part of the Clavien–Dindo classification [10]. Investigating nearly 6000 elective hysterectomies for benign and malignant causes, oncological surgery was found to have significantly more intraoperative complications (10% versus 3%) and reoperations (4% versus 2%) compared to surgery for benign causes [15].

Compliance and completeness are surrogate parameters. We were not present in the OR. It is possible that the security routine was followed but the checklist not completed, and it is possible that the security routine was NOT followed, but the checklist was still ticked off. We regard the latter as highly unlikely.

13 of 52 (25%) surgeries during the night shifts were revisions due to postoperative complications after elective surgery performed during day shifts. This needs to be considered when thinking about complication rates during day and night shifts. To investigate whether postoperative complications after revisions occur at a different rate than after primary surgery was not an aim of this study.

The observation that patients were younger and operation time was less during night shifts can be explained by the fact that routine surgeries including oncologic procedures and pelvic organ floor surgery are performed in older patients per se, whereas urgent procedures during the night are more often performed for miscarriage, extrauterine pregnancy and pelvic inflammatory disease.

The high number of team changes can be explained by our institution being an educational center. The majority of team changes occurred because the supervised intern handed over the operation to the supervising consultant doctor.

A strength of our study is that it was performed at a university hospital with a wide range of surgeries including infertility, oncology, and uro-gynecology.

Our study has some limitations. It was conducted in only one setting and in a brief period of time; therefore, the results might not be applicable to other settings throughout the country. Moreover, the study relies on data from the patients’ medical records and validation of checklist utilization is not presented. The authors did not make direct observations during the procedures.

It remains unclear whether an incomplete checklist is because somebody missed to tick the certain position or the OR team missed to complete not ticked questions.

Furthermore, surgeries during day shifts were mostly elective, whereas during night shifts a substantial part of revision operations were performed, which leads to difficulties when comparing the complication rates between day and night shifts.

To sum up, this study reports a high compliance rate with mandatory checklist use, but low percentages of completed checklists. During night shifts, checklists were less often available compared to day shifts.

Compliance with the WHO surgical safety checklist is seemingly high, but completeness remains low and there is room for improvement of the safety culture in this setting.

## Supplementary Information

Below is the link to the electronic supplementary material.

Supplementary file1 (PDF 11888 KB)

## Data Availability

The data presented in this study are available on request from the corresponding author. The data are not publicly available due to ethical restrictions.
