# Supplementary material for: Comparing compliance with the WHO surgical safety checklist and complication rates in gynecologic surgery between day and night shifts
Source: Arch Gynecol Obstet. 2022 May 27;306(4):1101–6. doi: 10.1007/s00404-022-06599-w (PMC9470616; doi:10.1007/s00404-022-06599-w)
Supplement: Supplementary file 1 — Supplementary file1 (PDF 11888 KB) [file 404_2022_6599_MOESM1_ESM.pdf]

Sicherheitscheck

⇨ durchgeführt vom chirurgischen Personal vor Transport zum OP

- O *Operationsart und –stelle/ Markierung?* C
- O *Operationseinwilligung ausgefüllt?* C
- O *Blutkonserven bestellt?* C
- O *Covid 19 Abstrich nicht älter als 48 Stunden* C
- O *Inhaltliche Kontrolle OP-Programm?* C

Durchgeführt von
Datum und Uhrzeit ______________

1 Sign In

⇨ unmittelbar vor Einleitung des Anästhesieverfahrens

- O *PatientInnenidentität geprüft?* (BP), A, (AP, K), OP
- O *Operationsart/ –stelle lt. OP-Programm?* A, OP, (K)
- O *Implantierte Medizinprodukte?* A, OP (K)
- O *Welche Allergien sind bekannt?* A, AP, (K), OP
- O *Equipment analog zum geplanten Eingriff vorhanden?* OP, A, AP, (OPA, K)
- O *Besondere Hygienemaßnahmen Erforderlich(Covid 19?)?* A, (AP, K), OP
- O *Antibiotika – Prophylaxe durchgeführt?* A
- O *PatientInnenbefunde vollständig/ vor Ort?* A
- O *Anästhesieeinwilligung ausgefüllt und vor Ort?* A
- O *Schwieriger Atemweg/ Aspirationsrisiko?* A
- O *Monitoring angelegt und funktionsfähig incl. allgemeiner Anästhesiecheck? (EKG, Pulsoximetrie, Blutdruck)* A, AP
- O *Blutkonservencheck erledigt?* A, AP
- O *Offene Fragen* A, OP, AP, (K), OPA

2 Team Time Out

⇨ unmittelbar vor Beginn des Eingriffs

- O *Kennen Sich die Teammitglieder (Vorstellung)?*
- O *PatientInnenidentität geprüft?* A, C, (AP, K), OP
- O *Operationseinwilligung ausgefüllt und vor Ort?* C
- O *Operationsart und -stelle, Operationsseite?* C
- O *Equipment überprüft und einsatzbereit?* A, AP, C, (K), OP
- O *Medizinische Besonderheiten zu erwarten?* A, C
- O *Geplante Blutkonserven adäquat?* A, C
- O *Personenschutzmaßnahmen erforderlich? (Röntgen, Hygiene, ...)* A, AP, C, (K), OP, OPA
- O *Offene Fragen* A, C, OP, (K), AP, OPA

PatientInnenetikett

Bitte beachten Sie die Arbeitsanweisung „OP-Sicherheitschecklisten anwenden“

3 Sign Out

⇨ unmittelbar vor Eingriffsende

- O *Welcher Eingriff wurde durchgeführt?* C
- O *Tücher und Instrumente komplett?* OP
- O *Gewebeproben korrekt beschriftet?* OP, OPA
- O *Informationen für die postoperative Betreuung der PatientIn* A, C
- O *Wo wird die PatientIn postoperativ betreut?* A C
- O *Offene Fragen* A, AP, C, OP, (K), OPA

Legende:
Personen, die für die Beantwortung der jeweiligen Fragen zuständig sind:

A	AnästhesistIn
AP	Anästhesie-Pflege
C	ChirurgIn
OP	OP-Pflege
OPA	OP-AssistentIn
K	KardiotechnikerIn
BP	Erwachsene Begleitperson und/oder PatientIn
()	Klammer = wenn anwesend

OP-Datum: ______________
